# Difficult Dental Prosthesis

**Published:** 1892-12

**Authors:** I. Douglas

**Affiliations:** Romeo, Mich.


### Difficult Dental Prosthesis.

BY I. DOUGLAS, D.D.S., ROMEO, MICH.

Read before the Michigan State Dental Society.

A very peculiar case came to me in August, 1852. A peddler had been wearing a plate which was never satisfactory, and was finally obliged to discard it altogether. His four upper incisors and all of the osseous structure which had supported them, were entirely gone, even up to the floor of the nasal passages, and arching as far back as the posterior approximate surface of the first bicuspids. The cuspids, however, were in place. In line with the bicuspids there was an oval opening into the nasal cavity, three-eights of an inch long from front to back, and one-fourth of an inch wide. In line with the twelfth-year molars, a little to the right of the center, there was another opening into the nasal cavity, slightly smaller than the first. Without these two openings being closed he was obliged to hold his nose in order to articulate so as to be understood.

As the second bicuspids on either side had been filed away preparatory to filling, there was opportunity for clasps that would rarely show.

On account of the difference in price, he chose a silver plate in preference to gold. The impression was taken in wax, and the model and dies gotten up in the usual manner. In swaging up the plate to fit the roof of the mouth, it was allowed to extend backward to cover the smaller of the two abnormal openings, and forward to the line where the soft tissues began to droop to the lip, the edge of the plate turning downward slightly.

The plate was then placed upon the zinc die, and built in with plaster to make it correspond with the form of the natural gums. An impression of these newly constructed parts was taken, new dies were prepared, and a second plate was swaged, the edges of which should come in contact with the first. These two plates were soldered together, the space between them corresponding with the missing parts of the mouth.

The clasps were now fitted to the selected teeth, and plate and clasps put in place. Impressions were taken, on each side separately, of the relative position of plate and clasps, to aid in soldering them together. A piece of warmed wax was pressed against the plate and clasps, then chilled with a bit of wet cotton, and loosened by a quick motion so as not to bend the wax. The plate and clasps, after being removed from the mouth, were placed in position and carefully imbedded in a mixture of one part plaster and two parts sand.

After soldering the clasps to the plate, the teeth were ground and backed in the usual way. When the teeth were to be soldered to the plate, a little borax and solder were placed on the seam between the two plates, as a precaution, lest the reheating should cause some of the first solder to flow away.

After the patient had worn this plate four weeks, he reported that it was perfectly satisfactory.

Case 2. In 1882, a traveling German called, having lost his two upper central incisors, and one lateral, also the right sixth-year molar. There was an opening through the palatine arch, into the nasal cavity, oval in shape, midway between the cuspids.

There would be no difficulty in making this job with clasps; but he had been wearing a plate with clasps, and, as a result, had lost one molar; and both of the bicuspids on the other side were badly decayed from being filed to make room for clasps; consequently he wanted no more clasps. Being informed that he could have a job without clasps that would keep its place except in case of sneezing, coughing or vomiting, he replied that he was an inveterate sneezer. So he finally decided to allow a gold clasp around the right twelfth-year molar to guard against its being thrown out of the mouth by spasmodic action. Accordingly, the plate was made with two air-chambers, one on either side of the opening; the air chambers, front and back, approaching each other to within one-eighth of an inch. A portion of the upper side of the plate was allowed to extend upward nearly through this abnormal opening, round the top of which just above the bone, was a very slight rim. This was to prevent the air and the secretions from the nasal mucous membrane from passing downward as readily as they would otherwise.

Before fitting this plate, the ends of the clasp were bent slightly away from the tooth. When the plate seemed to fit all right, the clasps were again put in place. After a trial of a few weeks, the patient reported the denture satisfactory.

Case 3. About 1852, a Mr. S. had Polypus in the nose. A physician gave him calomel to use as a snuff to destroy the abnormal growth, which it did ; but it also destroyed the entire nose and upper lip. In due time he went to the surgeons in Ann Arbor, who took material from his cheeks to make an upper lip. A year or two later, he returned, and they took material from his forehead and made a nose. The improvement in his appearance was very marked; for, instead of an unsightly black looking hole, he had quite a respectable looking nose. But, being without bony or cartilaginous support, it flattened down after some years.

About 21 years ago, this man came to me, having lost his four upper incisors and one cuspid. I made him a denture; but in order to do this, it was necessary to cut off each side of an upper impression tray, till it measured only one and three-eighths of an inch in the widest part; for when he opened his mouth as widely as he could, the opening was only a trifle over an inch across and around.

At the time of making this partial plate, I informed him that unless he persevered in stretching his lips, he could never have a full set of teeth ; that this would be necessary, as he was going to lose all of his natural ones.

This same man came to me a few months ago for a full set of upper teeth. Measurement with inside calipers showed that an impression tray must be at least two and one-fourth inches wide by two inches long. A No. 4, S. S. White tray was given to the patient, and, after repeated trials, he with difficulty succeeded in putting it into his mouth, after bending inward both lateral sides.

To add to the difficulties of this case, the passage between the throat and posterior nares is entirely closed ; also, there are no angles to his mouth. In taking the impression, it was necessary to use as little plaster as possible and before removing the impression, the surplus plaster had to be trimmed off from each side; and even then the extreme tension started the blood. There was no further difficulty in completing this set. The back teeth were set as far outward as seemed prudent. The patient had to put the plate in sidewise, and turn it around afterward. But the work was satisfactory, and the patient returned in a few days for a lower set.

Here the difficulties seemed insurmountable. I could not introduce a lower impression tray to ascertain if it were the right size. I went to our machine shop and borrowed a pair of two-jointed, double-setting, inside pliers, with these, I ascertained that a No. 3, S. S. White tray had to be spread to make it wide enough, and the mouth only about an inch and one-fourth across. The tray must be cut in two, and the impression taken in two sections, or made so afterward. Shaping a piece of wax to the upper surface of the handle of the tray, and letting it extend a little on to the tray proper, and using this for a pattern to make a mold, a piece of britannia was cast to fit the handle and tray. The handle itself was strengthened by adding solder to the under side. Four holes were then drilled through the new plate and tray handle, two on each side, and four guide-pins were fitted to the holes. The tray was next sawed in two through the entire handle, while the britannia plate was left in one piece, and served to hold the two pieces of the tray together and secure by the pins.

In taking the impression, the two halves of the tray were filled with plaster and introduced into the mouth separately, then locked together by the britannia plate and guide-pins and pressed down to its place.

Some modeling composition was taken from a basin of hot water and fitted to the underside of the handle, and then cooled with a napkin wet in cold water. The plaster impression was raised from the jaw, and the britannia plate removed; leaving the modeling composition to hold the two sections of the tray together. A ribbon saw was next inserted between the two portions of the tray, and the plaster sawed part way and then broken and removed from the mouth in two parts. Thus a perfect model was obtained.

It was necessary to strenghten the wax trial-plate by three wires, one on either edge of the plate in addition to one on the lingual side of the ridge, just out of the way of the teeth, for the patient was obliged to put the plate into his mouth sidewise and then turn it around. The bite was secured by putting soft wax on the trial-plate. When the plate was ready to fit, in order to adjust the occlusion, a thin sheet of wax was pressed over the grinding surfaces of the side teeth, for the patient to bite on. When the occlusion was nearly exact, the indications for its completion were obtained by placing a strip of carbon paper between the grinding surfaces while in the mouth.

On account of being obliged to raise the lower lip so very high to meet the upper in articulation, the soft tissues in this case, raising nearly to the top of the gums in front; it was necessary to cut away the plate to prevent its excoriating. This patient now has a set of teeth which he prizes very highly.

A case of amateur dentistry, which is simply amusing, will complete the list.

A lady had been wearing a plate with two front teeth. One morning she dropped them on the floor and stepped on them, breaking off both the teeth. She had engaged to go with her best fellow to a party the following evening. A dentist would not mend them in time, as she lived some distance in the country. She could not go without them; yet go she must. She was in a dilemma.

Fastening the pieces of rubber together with wax, she had a model to work from. She would carve out a set of teeth for herself. What material could she use? First she tried a white turnip. Discarding that, she took a yellow baga turnip, which was of a firmer texture and a better color. And with this baga turnip and a pocket-knife, she actually carved out a plate with teeth attached, the whole thing the size and shape of the original "store teeth." And so successful was her work that she wore this turnip-plate to the party without detection. Soon after she carved a second set which she wore once. After that she again applied for professional services.

